# Fellows’ Hypo Phosphites

**Published:** 1880-05

**Authors:** 


### FELLOWS' HYPO-PHOSPHITES.

THIS preparation combines phosphorus and strychnia in a very palatable and reliable form, especially adapted to the treatment of nervous affections, as well as a valuable auxiliary in bronchial and pulmonary diseases. It possesses also the property of forming an emulsion with cod liver oil, making a convenient combination in diseases in which these important therapeutic agents are indicated.

The sentiment of the profession in this vicinity is decidedly favorable to this preparation, and we do not hesitate to recommend it.

